# Screening of *E. coli* β-clamp Inhibitors Revealed that Few Inhibit *Helicobacter pylori* More Effectively: Structural and Functional Characterization

**DOI:** 10.3390/antibiotics7010005

**Published:** 2018-01-11

**Authors:** Preeti Pandey, Vijay Verma, Suman Kumar Dhar, Samudrala Gourinath

**Affiliations:** 1 School of Life Sciences, Jawaharlal Nehru University, New Delhi 110067, India; preet.satya@gmail.com; 2 Department of Bioscience and Biotechnology, Banasthali University, Rajasthan 304022, India; 3 Special Centre for Molecular Medicine, Jawaharlal Nehru University, New Delhi 110067, India; vijayscmmjnu@gmail.com (V.V.); skdhar2002@yahoo.co.in (S.K.D.); 4 Department of Microbiology, Central University of Rajasthan, Kishangarh 305801, India

**Keywords:** DNA replication, surface competition assay, β-clamp, *E. coli* inhibitors, structure, screening

## Abstract

The characteristic of interaction with various enzymes and processivity-promoting nature during DNA replication makes β-clamp an important drug target. *Helicobacter pylori* (*H. pylori*) have several unique features in DNA replication machinery that makes it different from other microorganisms. To find out whether difference in DNA replication proteins behavior accounts for any difference in drug response when compared to *E. coli*, in the present study, we have tested *E. coli* β-clamp inhibitor molecules against *H. pylori* β-clamp. Various approaches were used to test the binding of inhibitors to *H. pylori* β-clamp including docking, surface competition assay, complex structure determination, as well as antimicrobial assay. Out of five shortlisted inhibitor molecules on the basis of docking score, three molecules, 5-chloroisatin, carprofen, and 3,4-difluorobenzamide were co-crystallized with *H. pylori* β-clamp and the structures show that they bind at the protein-protein interaction site as expected. In vivo studies showed only two molecules, 5-chloroisatin, and 3,4-difluorobenzamide inhibited the growth of the pylori with MIC values in micro molar range, which is better than the inhibitory effect of the same drugs on *E. coli*. Therefore, the evaluation of such drugs against *H. pylori* may explore the possibility to use to generate species-specific pharmacophore for development of new drugs against *H. pylori*.

## 1. Introduction

There are certain pathogens that are affecting the human population worldwide. *Helicobacter pylori* (*H. pylori*) is one of them that infects around 50% of the world’s population by causing peptic ulcer, gastritis, and gastric cancer [1]. For the conditions that are associated with *H. pylori*, eradication of infection using antibiotics as well as acid-suppressing medication is highly effective clinical intervention. Current procedure is becoming difficult because of the increasing cases of the antibiotic-resistant strains [2,3]. These resistances are mainly associated with mutation in their target enzyme/protein. Therefore, there is an emergent need to find out promising drug targets that help in eradication of such disastrous pathogen.

The DNA replication machinery offers several important and interesting drug targets [4,5]. Among them one such target is β-clamp. β-clamp, a part of DNA pol III holoenzyme is very crucial protein as it is involved in myriads of steps during DNA replication. It is the key protein that increases the processivity of many important enzymes and complexes by interacting with them. β-clamp prevents the polymerase from dissociating, while polymerase’s rapid movement along the DNA molecule. Apart from several DNA polymerases, pol I [6], pol II [7,8], pol IV [9], pol V [10], β-clamp interacts with various proteins such as mismatch repair proteins MutL and MutS [6], DNA ligase [6], and the DnaA-related protein Hda [11].

Biological active form of β-clamp is dimer and each monomeric unit consists of three domains, where the protein-binding site resides in between domain II and III. This interaction site is divided into two subsites and all β-clamp interacting partners bind to these subsites [12,13,14,15]. Usually, the proteins that interact with β-clamp in bacteria contain consensus sequence with five or six residues, with the consensus amino acid sequence of “ZXSLF” to bind to the binding pocket of β-clamp [16] where Z represents any amino acid which is hydrophilic in nature while X represents any small hydrophobic residue. This protein-protein interaction site has been target for drug development in different organisms. Recently, we have reported diflunisal a FDA approved drug that binds to this site and can inhibit the growth of *H. pylori* at a micromolar concentration [17]. Many inhibitors and drugs for *E. coli* β-clamp have been identified but regardless of their similar structure, they are not equally effective in other organism [18]. Therefore, drug effective for one organism may not show the same effect on the other. *H. pylori* shows extreme genetic variability and allelic diversity because of intraspecific recombination and mutations [19]. It has been found that many proteins as well as their functions vary in *H. pylori* from that of other bacteria, especially from *E. coli* like helicase and primase strong interaction [20,21], loading of helicase is different from that in *E. coli* [22,23].

In a previous manuscript, we found some differences at DNA binding as well as protein binding regions in native Hpβ-clamp when compared to other β-clamp structures from various organisms [16]. This made us think whether there is any difference in binding pattern of inhibitors or the drugs that are already known to inhibit *E. coli* β-clamp could inhibit *H. pylori* β clamp also. In order to find that, we examined drugs and inhibitors that are known to bind *E. coli* β-clamp, albeit their IC_50_ is in millimolar range [18]. On the basis of docking score, five molecules were selected for in vitro and in vivo studies. All of the five molecules showed competitive binding with ligase and three of them were co-crystallized and determined the complex structure with Hpβ-clamp to show that they bind to the ligase binding site/protein-protein interaction site. Among these three, two molecules 5-chloroisatin and 3,4-difluorobenzamide showed inhibition of *H. pylori* growth with micromolar IC_50_ values.

## 2. Results and Discussions

### 2.1. Screening of E. coli β-clamp Drugs/Inhibitors

The structures of *E. coli* β-clamp in complex with some of its inhibitors have already been reported [18,24,25,26]. In order to check the extent to which these inhibitors would also inhibit Hpβ-clamp, we docked all of them against Hpβ-clamp. Finally, we shortlisted five of these molecules from the PDB depending on their docking scores and availability; these molecules were 5-chloroisatin (C1) (PDB id: 4N95), 6-nitroindazole (C2) (PDB id: 4N96), (S)-carprofen (C3) (PDB id: 4MJR), 5-nitroindole (C4) (PDB id: 4N97), and 3, 4-difluorobenzamide (C5) (PDB id: 4N94) (Table S1).

### 2.2. Competitive Inhibition Using Surface Competition Assay

**Figure 1:**
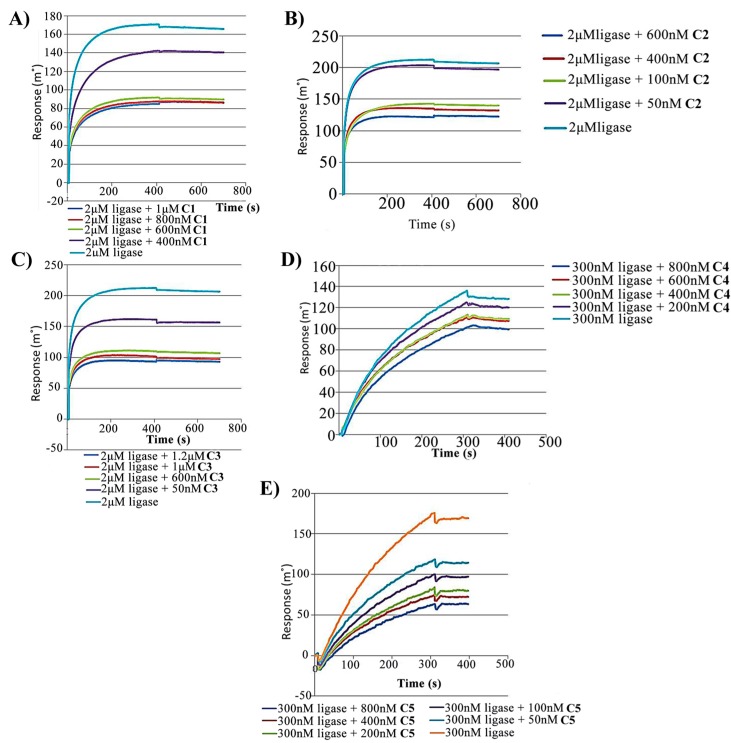
SPR sensorgram. SPR sensorgram showing surface competition assay between HpDNA ligase and the small molecules. A qualitative analysis of the in vitro competition between DNA ligase and small molecules (present in solution) for binding to Hpβ-clamp (immobilized on the chip surface) was carried out. For the small molecules (**A**) 5-chloroisatin (C1); (**B**) 6-nitroindazole (C2) and (**C**) (S)-carprofen (C3), a mass of ~6 ng of Hpβ-clamp gets immobilized on the chip surface while for (**D**) 5-nitroindole (C4) and (**E**) 3,4-difluorobenzamide (C5), a mass of ~4 ng of Hpβ-clamp gets immobilized on the chip surface. The concentration of ligase was kept the same throughout each experiment with a small molecule. As the concentration of the small molecule was increased, the SPR response decreased.

Due to small size and low molecular weight of the shortlisted molecules we were unable to predict the interaction between them and Hpβ-clamp using the simple SPR binding technique. Therefore, we did a qualitative analysis by choosing an advanced approach of surface competition assay where the sensorgram response decreases with increasing concentration of the analyte molecule. This approach is basically based on competition between two analyte molecules that compete for binding to the same ligand. Here, Hpβ-clamp was used as ligand, HpDNA ligase (a natural binder of β-clamp [16]) was used as analyte 1 and drugs/inhibitor molecules were used as analyte 2, similar to the protocol reported in Pandey et al., 2017. Since mixture of both the analytes, DNA ligase and drugs/inhibitor were passed through the SPR chip, the summation of both analyte contribution was the measured response in this technique. There was an inverse relationship between magnitude of response obtained and amount of small molecule analyte in the sample [27]. Since it was a competition assay so for binding to β-clamp, the drug/inhibitor molecules should compete with ligase. The sensorgram showed a decreased response as we increased the concentration of small molecule (Figure 1), only because now more and more protein binding site is occupied by low molecular weight analyte i.e., drug/inhibitor molecules (which has negligible weight), and thereby blocking the binding of high molecular weight ligase (which was responsible for detectable signals in sensorgram). All five shortlisted molecules showed competitive binding to β-clamp. The inhibitory effect of each of the small molecules is shown by a continuous decrease in the sensorgram response withan increasing small molecule concentration.

### 2.3. Binding Pattern Analysis in Complex Crystal Structures

**Figure 2:**
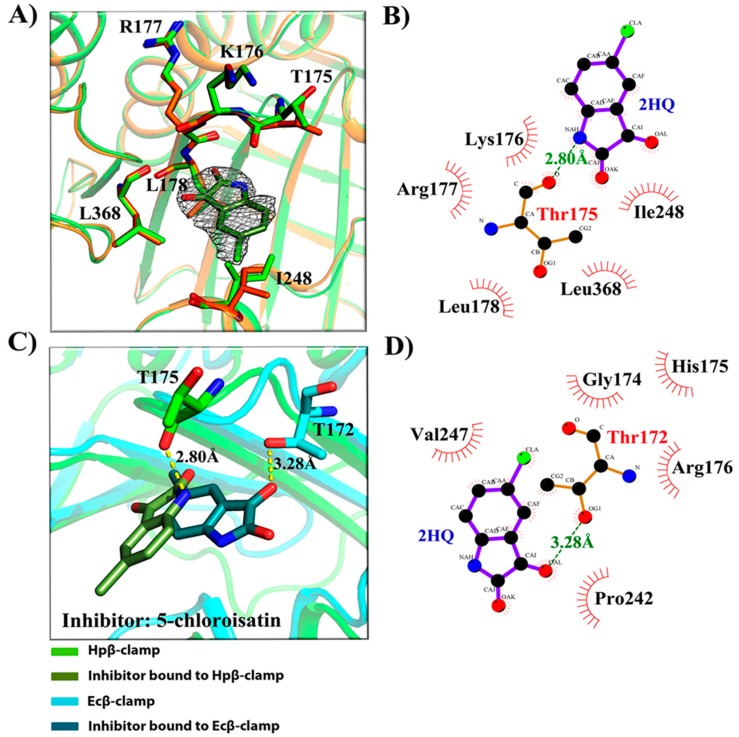
5-chloroisatin interaction with Hpβ-clamp. (**A**) 2Fo-Fc map, contoured at 1σ, of 5-chloroisatin bound to Hpβ-clamp (PDB ID: 5G4Q). The alignment of the structure of the complex (green) with that of the native (orange) did not yield significant differences in the orientation of interacting residues except for I248; (**B**) Ligplot of the Hpβ-clamp structure near the bound 5-chloroisatin, showing the predominantly hydrophobic interactions between the protein and the inhibitor. T173 of Hpβ-clamp did form an H-bond with the inhibitor molecule; (**C**) Structural alignment of Hpβ-clamp (green) and Ecβ-clamp (cyan) complex with ligand 5-chloroisatin. T172 of Ecβ-clamp makes H-bond with ligand while T175 of Hpβ-clamp makes H-bond with the ligand (PDB ID: 4N95); (**D**) Ligplot of Ecβ-clamp complex with 5-chloroisatin showing the types of interactions between them.

**Figure 3:**
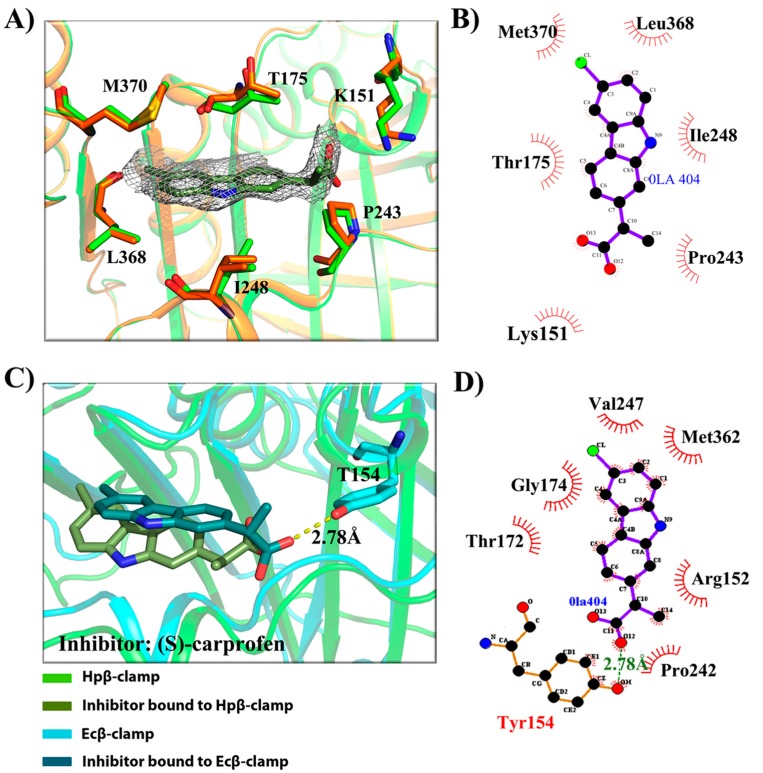
(S)-carprofen interaction with β-clamp. (**A**) 2Fo-Fc map, contoured at 1σ, of (S)-carprofen bound to Hpβ-clamp (PDB ID: 5FXT). The alignment of the structure of the co-crystal of Hpβ-clamp (green) and the inhibitor (olive) with that of the native Hpβ-clamp (orange) showed almost same orientation of interacting molecules in both structures; (**B**) Ligplot of the Hpβ-clamp structure near the bound (S)-carprofen, showing the hydrophobic interactions between the protein and the inhibitor; (**C**) Structural alignment of *H. pylori* (green) and *E. coli* β-clamp (cyan) complex with ligand (S)-carprofen. T154 of Ecβ-clamp makes H-bond with the ligand apart from other hydrophobic interactions while Hpβ-clamp and ligand interactions are dominated by hydrophobic interactions; and, (**D**) Ligplot of Ecβ-clamp with bound ligand showing its various interactions with ligand (PDB ID: 4MJR).

**Figure 4:**
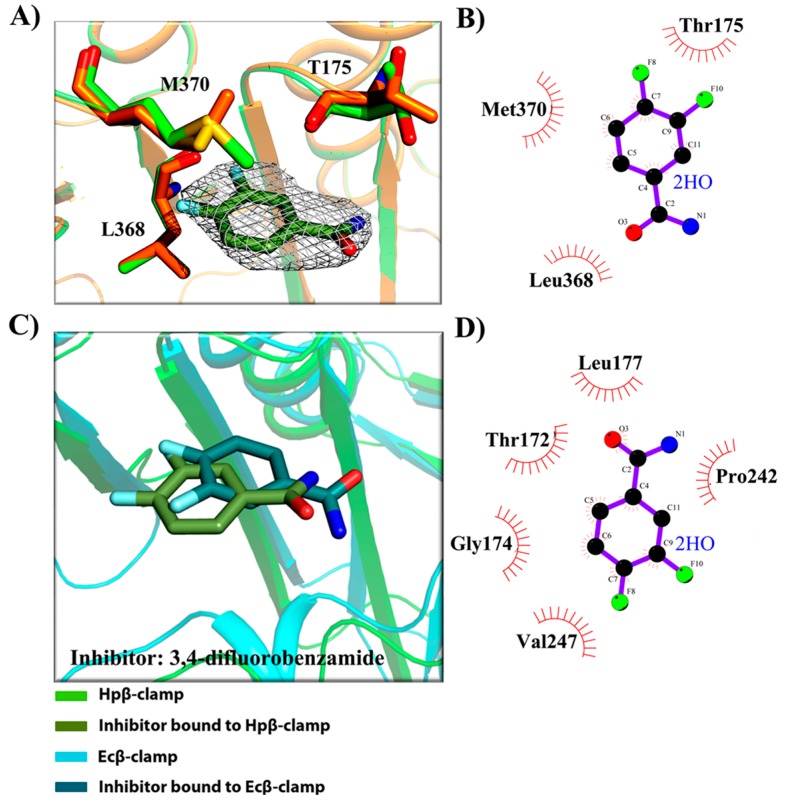
3, 4-difluorobenzamide interaction with β-clamp. (**A**) 2Fo-Fc map, contoured at 1σ, of 3, 4-difluorobenzamide in complex with Hpβ-clamp. The alignment of the structure of the co-crystal of Hpβ-clamp (green) and the inhibitor (olive) with that of the native Hpβ-clamp (orange) showed differences in the orientations of residues T175, M370, K176 and I248 between the co-crystal and native structures; (**B**) Ligplot of the Hpβ-clamp structure near the bound 3, 4-difluorobenzamide, showing the hydrophobic interactions between the protein and the inhibitor; (**C**) Structural superimposition of Hpβ-clamp (green) and Ecβ-clamp (cyan) complex with ligand 3, 4-difluorobenzamide. In both the cases, the contacts are dominated by hydrophobic interactions however the orientation of ligand molecule is differentiated by a rotation of 180 degree; and, (**D**) Ligplot of Ecβ-clamp bound to ligand showing the hydrophobic interactions nearby (PDB ID: 4N94).

After confirmation of inhibitory activity in surface competition assay, we tried the co-crystallization of those inhibitors with Hpβ-clamp, and three of them were successfully co-crystallized with Hpβ-clamp. These were 5-chloroisatin (C1) (PDB id: 5G4Q), carprofen (C3) (PDB id: 5FXT) and 3, 4-difluorobenzamide (C5) (PDB id: 5FVE) (Figure 2, Figure 3 and Figure 4). All of these crystals yielded clear electron density for the ligand molecules with good occupancy, except for 5-chloroisatin. The occupancy of 5-chloroisatin was quite low. Moreover, based on data obtained from the soaked crystals, the temperature factor of this inhibitor was high when compared to the average temperature factor of the protein. Some of the following points regarding the binding pattern here were derived from inspection of the co-crystal structures with various inhibitors.

**Figure 5:**
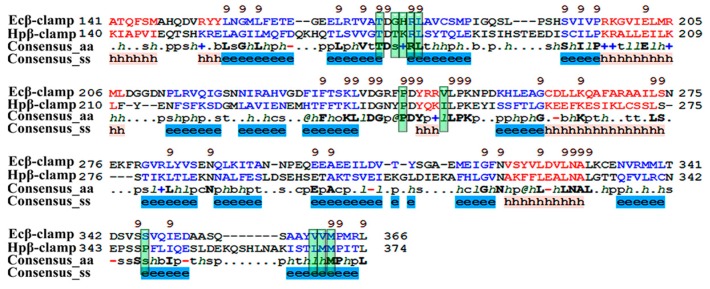
Structure-based sequence alignment. β-clamps of *H. pylori* and *E. coli* were compared using structure based sequence alignment. The ligand-interacting residues are highlighted (in green box). In each block, the first line shows conservation indices for positions with a conservation index above 5. The secondary structure prediction is shown in color red (alpha-helix) and blue (beta-strand). The last two lines show consensus amino acid sequence (consensus_aa) and consensus predicted secondary structures (consensus_ss). Consensus predicted secondary structure symbols: alpha-helix:h; beta-strand:e. Consensus amino acid symbols are: conserved amino acid are in uppercase and bold letter; aliphatic (I,V, L): l; aromatic (YHWF); hydrophobic (W,F,Y,M,L,I,V,A,C,T,H): h; alcohol (S,T):o; polar residues (D,E,H,K,N,Q,R,S,T): p; tiny (A,G,C,S):t; small (A,G,C,S,V,N,D,T,P):s; bulky residues (E,F,I,K,L,M,Q,R,W,Y):b; positively charged (K,R,H):+; negatively charged (D,E): -; charged (D,E,K,R,H):c.

(a) Binding site of β-clamp is conserved in all bacterial species: The ligand-binding site of β-clamp consists of hydrophobic amino acid residues that have been found to be fairly conserved but not identical in the β-clamps of the various species reported so far. As described in introduction, two protein-binding subsites located near each other, subsite I and subsite II, have been identified in β-clamp (Figure S1). All of the proteins and peptides that have been observed to interact with β-clamp in structures that are deposited in the PDB have been shown to bind to the region of β-clamp encompassing subsites I and II as seen in Ecβ-clamp [9,24,28] and in our earlier studies on ligase peptide-bound Hpβ-clamp [17]. Inspection of the crystal structures of the inhibitor-Hpβ-clamp complexes (PDB id: 5g4q, 5FXT, 5FVE) and inhibitor-Ecβ-clamp complexes (PDB id: 4N95, 4MJR, 4N94) revealed the inhibitor-interacting residues of Hpβ-clamp correspond to those of Ecβ-clamp [9,24,28], suggesting the importance of these residues in the protein-binding cleft (Table S2) (Figure 2, Figure 3 and Figure 4). These residues, especially those corresponding to Thr173, Thr175, Pro243, Ile248, Met370 of subsite I, are quite conserved in β-clamp and are ligand binding residues. Most of these residues also observed to interact with the ligase peptide, clearly indicating that these small molecules will compete with other proteins, like ligase, to bind to β-clamp.
(b) All of the inhibitors occupy subsite I of the protein interaction site: In the crystal structure of Hpβ-clamp complexed with the FIRSLF peptide from HpDNA ligase (PDB ID: 5FRQ), the peptide was observed to occupy both subsites I and II, like other clamp-interacting proteins/peptides from *E. coli* [16]. Residues Leu360 and Phe361 of this ligase peptide were observed to be buried deep in the Hpβ-clamp cleft and to make hydrophobic contacts with Hpβ-clamp subsite I residues Thr173, Lys176, Ile248, Pro347, Leu368, and Met370 (PDB ID: 5FRQ). Moreover, all of the inhibitors we crystallized with Hpβ-clamp also bound to subsite I. The ligplots of the Hpβ-clamp residues contacting each inhibitor are shown in Figure 2, Figure 3 and Figure 4. The inhibitor-interacting residues were found to be the same as those that were observed to interact with HpDNA ligase (Table S2). All of these inhibitors were observed to interact with most of the residues of subsite I that formed contacts with the ligase peptide. A comparison of the ligand-bound Hpβ-clamp structure with the native Hpβ-clamp structure yielded no significant difference in the positions of the ligand-interacting residues (Figure 2, Figure 3 and Figure 4) except for Lys176 and Met370, which moved a little bit from their native positions to firmly bind the inhibitor. Thr175 of Hpβ-clamp was also observed to make a hydrogen bond with the inhibitor 5-chloroisatin.
(c) Comparison of co-crystal structures of *H. pylori* and *E. coli* β-clamp: The comparison of co-complex structures of *H. pylori* and *E. coli* with various inhibitors showed some critical differences in the binding pattern. In case of 5-chloroisatin (Figure 2), the Thr175 of Hpβ-clamp makes hydrogen bond with ligand however that in Ecβ-clamp is made by Thr172 in the neighborhood. Out of various hydrophobic contacts between the β-clamp and inhibitor, three of them are common in both *H. pylori* and *E. coli* β-clamp. These residues include Ile248, Lys176, Arg177 in Hpβ-clamp, and Val247, His175, Arg176 in Ecβ-clamp. In case of (S)-carprofen (Figure 3), Thr154 of Ecβ-clamp makes H-bond with the ligand along with other hydrophobic contacts however in *H. pylori* the contact between β-clamp and ligand is favored by only hydrophobic interactions. Five of these interactions are common in both, which includes residues Lys151, Thr175, Pro243, Ile248, Met370 in Hpβ-clamp and Arg152, Gly174, Pro242, Val247, Met362 in Ecβ-clamp. In case of 3, 4-difluorobenzamide (Figure 4), the interaction between β-clamp and ligand in both *H. pylori* and *E. coli* is dominated by hydrophobic interactions. Among them, three of these interactions are common in both the clamps, Thr175, Pro243, Ile248 in Hpβ-clamp and Gly174, Pro242, Val247 in Ecβ-clamp, respectively. All of the ligand-interacting residues of Hpβ-clamp as well as Ecβ-clamp are tabulated (Table S2). The structure-based sequence alignment of Hpβ-clamp and Ecβ-clamp is shown in Figure 5 with highlighted ligand-interacting residues, which shows that the mode of interaction between inhibitor/ligand and β-clamp of both organisms are almost same.
(d) Comparison of the co-crystal Hpβ-clamp structures with docked structure: In all of the docked structures, like in crystal structure, the inhibitors were found to be present in the same pocket (Figure S2). The orientation of the 5-chloroisatin inhibitor in the docked structure did differ from that in the crystal structure (Figure S2), but this difference did not affect much the structure of the binding site. Hpβ-clamp residue Thr173 form hydrogen bond with every docked inhibitor, while residue Lys247 hydrogen bonded the docked inhibitors (S)-carprofen. In contrast, in the actual crystal structure, only 5-chloroisatin was observed to make a hydrogen bond with Thr175 (Figure 2B). In every case, hydrophobic contact dominates the interactions between the protein and inhibitor. Interestingly, all of the docked ligands and ligands in co-crystal structures bound in the same pocket with similar hydrophobic interactions, but the hydrogen bond pattern did not match.

### 2.4. Antibacterial Activity of Hpβ-clamp Inhibitors

**Figure 6:**
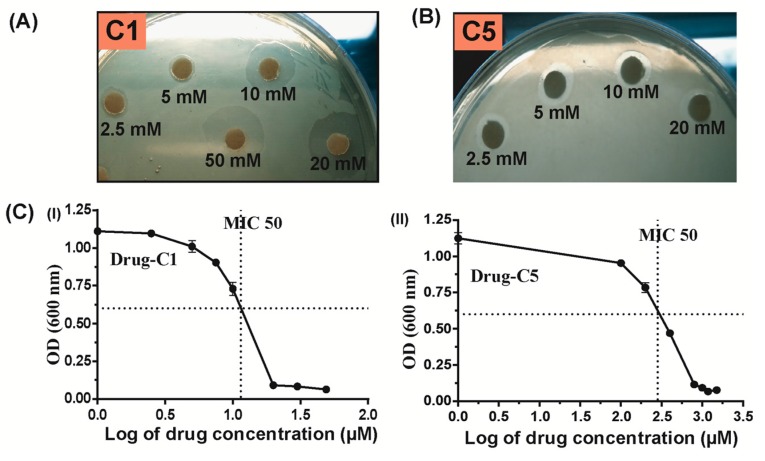
Antimicrobial activities of different drugs against *H. pylori*. (**A**) Anti-*H. pylori* activities of different drugs (**A**) 5-chloroisatin (C1) and (**B**) 3,4-difluorobenzamide (C5) determined by applying the disk diffusion method. Petri dish with drugs containing discs showed inhibition zones for bacterial growth; (**C**) Anti-*H. pylori* activities of drugs determined as minimum inhibitory concentrations (MICs) were obtained via the dilution method. The MIC of drug C1 and C5 are 18 µM and 824 µM, respectively. The experiments were performed in triplicates. Error bars show standard deviation of the mean (Mean ± SD).

The response of each drug (C1-C5) on *H. pylori* 26695 was evaluated by disk diffusion method [29]. Out of five drugs, C5 (3,4-difluorobenzamide) showed marginal inhibition, while drug C1 (5-chloroisatin) showed significant inhibition zone on BHI agar plate against *H. pylori* (Figure 6A). However, no clear inhibition zones were observed in BHI-agar plates in case of other three drugs (data not shown) suggested that these three drugs have no inhibition on growth of *H. pylori* (data not shown). The results suggested that these two drugs (C1 and C5) might have inhibited the function of Hpβ-clamp thus preventing the *H. pylori* growth in culture. As mentioned in Materials and Methods, the MICs of drugs C1-C5 were measured on BHI-broth culture of *H. pylori*. With an MIC of 18 µM drug C1 (5-chloroisatin) showed significant antimicrobial activity (Figure 6C). In contrast, the drug C5 (3, 4-difluorobenzamide) yielded an MIC of 824 µM. These results suggested that drug C5 (3, 4-difluorobenzamide) and especially drug C1 (5-chloroisatin) to be effective against *H. pylori.* Although all of the above drugs (C1–C5) bound to Hpβ-clamp and showed their inhibitory effects in surface competition assay, only a few of them showed an inhibition in antimicrobial assay. The drug C1 (5-chloroisatin) showed extra H-bond with Thr173 compared to other complex structures may be responsible for better binding and thus better inhibition. Additionally, differential uptake of drugs into the pathogen could be the other possible reason behind the difference in their inhibition activity. 

## 3. Material and Methods

### 3.1. Overexpression and Purification of Hpβ-clamp

As described in our previous work, Hpβ-clamp (Accession id: AJF10096) was cloned, expressed, and purified [16]. The recombinant plasmid of Hpβ-clamp was transformed into *E. coli* BL21 strain. The cells were grown initially at 37 °C till the O.D reaches to 0.6. Induction was given with 0.5 mM IPTG and then cells were incubated at 30 °C in shaker for 6 h. The cells were then harvested at 6000 rpm for 10 min. The cell pellet was suspended in lysis buffer consisting of 10 mM imidazole, 30 mM Tris (pH 7.5), 150 mm NaCl, 0.5% tween20, and 6 mM β-Mercaptoethanol. 0.3% lysozyme. After about 30–45 min of incubation with lysozyme the cells were sonicated and centrifuged at 13,000 rpm for 45 min at 4 °C. The Supernatant was collected and passed through Ni-NTA column, which was pre-equilibrated with buffer having 10 mM imidazole, 30 mM Tris (pH 7.5), 150 mm NaCl, and 6 mM β-Mercaptoethanol. After giving a wash with wash buffer containing 30 mM imidazole, elution was taken with buffer containing 150 mM imidazole, 30 mM Tris (pH 7.5), 150 mM NaCl, 6 mM βMe, and 30 mM arginine. This fraction was then concentrated using a centricon. The concentrated protein was further purified through gel filtration chromatography using Hi-Load G200 16/60 column (GE healthcare). Eluted fraction was checked on a 12% SDS-PAGE gel. The same buffers and procedure was followed to purify HpDNA ligase (Accession id: AJF10437).

### 3.2. In Silico Screening of Inhibitors

The ligase-binding site on Hpβ-clamp was targeted for screening inhibitors and ligand binding analysis following the protocol, as reported earlier [17]. For grid generation, the site where peptide from ligase was bound ^10^ (PDB ID: 5FRQ) was used. For docking Maestro was used that includes Schrodinger’s Protein Preparation Wizard [30], Schrodinger ligprep wizard, and glide. All known *E. coli* B-clamp inhibitors were chosen from Protein Data Bank (PDB) structures of their complexes with *E. coli* β-clamp with the hope that they would also efficiently inhibit the activity of Hpβ-clamp. Each molecule from *E. coli* β-clamp inhibitor was then prepared using Schrodinger’s Ligprep Wizard to generate a maximum of 34 conformations. Finally, docking was carried out using GLIDE [31,32,33], specifically its extra precision module after preparing the ligand and protein. Initially, we got number of molecules as a result of docking, but we were searching for those molecules, which were easily available. Therefore, we selected five molecules (Table S1) based on their GLIDE rankings as well as their availability. We then tried to co-crystallize these five inhibitor molecules with Hpβ-clamp. To test their inhibitory activities against Hpβ-clamp a biophysical experiment, as well as antimicrobial assay of these molecules, were carried out.

### 3.3. Surface Competition Assay

We performed a qualitative analysis using a surface competition approach, following the protocol as reported earlier [17]. On SPR sensor surface, β-clamp was immobilized and the analyte, high-molecular-weight DNA ligase was passed over the sensor surface. The DNA ligase was also mixed with low-molecular-weight compounds and was used as competitive analyte. The DNA ligase concentration was kept constant throughout this assay, however the competitive analytes concentration was increased in progressive injections. The sensorgram with different concentrations of analytes are shown in Figure 1. The flow cell 1 was used as a control surface for background subtraction, and in flow cell 2, β-clamp was immobilized. The SPR buffer used consisting of 10 mM HEPES pH 7.4, 150 mM saline-NaCl with 3 mM EDTA (HBS-EP) along with 2% dimethyl sulfoxide (DMSO) and 0.05% surfactant P20. All of the compounds were dissolved in DMSO and diluted in SPR buffer. The concentration of DMSO in buffer was matched with the DMSO amount in the experimental samples to limit bulk refractive index changes. The surface was regenerated between injections using high NaCl (0.5 to 1 M) solution wash.

### 3.4. Crystallization, Structure Determination and Analysis of the Complex Structures

Hanging drop vapor diffusion method was used to crystalize Hpβ-clamp in complex with the identified *E. coli* β-clamp inhibitors. The protein purified using gel filtration chromatography concentrated up to 6 mg/mL. Crystallization drop was put in 2:2 ratio, where 2 μL of protein was mixed with an equal volume of precipitant solution containing 20% *v*/*v* PEG MME 550, 0.1 M MOPS/HEPES-Na pH 7.3, 0.2 M ammonium citrate, 6% *w*/*v* PEG 20,000, 0.01 M MgCl_2_, and 0.01 M strontium chloride. 2 mM of small molecule (dissolved in DMSO) was added to the crystallization drop and equilibrated against 1 mL of the same precipitant in 24-well plates and incubated at 16 °C. The crystals appeared after about one week. The X-ray diffraction data were collected at DBT-ESRF beamline BM14 to a resolution of 1.9 Å–2.5 Å. To process and integrate the data HKL 2000 [34] was used. Native Hpβ-clamp structure was used as the search model to solve all of the co-crystal structures using the molecular replacement method and using the program Molrep in CCP4 [35]. Further, Refmac5 was used for refinement and COOT [36] was used for fitting the molecular structures into the electron density. Lastly, PROCHECK [37] was used to evaluate the stereochemistry of the model. The refinement statistics are listed in Table 1. The coordinates of complex structure are deposited with PDB IDs: 5G4Q (Hpβ-clamp complex with 5-chloroisatin, C1), 5FXT (Hpβ-clamp complex with carprofen, C3), and 5FVE (Hpβ-clamp complex with 3, 4-difluorobenzamide, C5).

### 3.5. Antimicrobial Test

(a) *H. pylori* strain and culture conditions: The *H. pylori* strain and culture conditions were used, as mentioned in our previous work [17]. In brief, Brain heart infusion (BHI) agar/broth medium was used to culture the *H. pylori* strain 26695. The agar/broth medium contains the antibiotics amphotericin B (8 mg/mL), trimethoprim (5 mg/mL), and vancomycin (6 mg/mL), 8% horse blood serum and 0.4% IsoVitaleX. The bacterial plates were incubated under microaerobic conditions (5% O_2_, 10% CO_2_) at 37 °C for 36 h and the broth cultures were incubated at 37 °C on a shaker operating at 110 rpm under microaerobic conditions (5% O_2_, 10% CO_2_).
(b) Anti-*H. pylori* susceptibility tests: To screen the susceptibility of *H. pylori* against drugs the Kirby-Bauer disk diffusion susceptibility test was used, as mentioned earlier [17,29]. In brief, 200 μL of the *H. pylori* suspension was spread evenly on 90 mm BHI-agar plates. Because of the slow growth of *H. pylori*, high inoculum was used for the disk diffusion method. Different concentrations of inhibitors (Sigma, St Louis, MI, USA) were inoculated (10 μL of each drug or DMSO as control) on 6 mm disks, which are placed on bacterial lawn surface. The disks were dried and incubated at 37 °C under micro-aerophilic conditions for about 3–5 days.
(c) Minimum inhibitory concentrations (MICs): The dilution method was applied to determine the MICs of drugs described earlier [17]. In brief, different concentrations of the drugs were used in 2 mL BHI broth that was inoculated with fresh *H. pylori* suspensions (20 µL; 1:100 dilution; initial optical density OD^600^ < 0.1). Further, the tubes containing broth and different concentration of drugs were incubated under microaerobic condition for 20 h at 37 °C on a shaker (110 rpm). After 20 h, the OD^600^ were taken and GraphPad software was used for analysis of MICs. OD^600^ measurements were also taken for the *H. pylori* suspension without drugs or with kanamycin (positive control) and with/without bacteria. All of the experiments were done in triplicate.

## 4. Conclusions

β-clamp is similar to proliferating cell nuclear antigen (PCNA) in higher organisms, including humans. It is required to maintain the fidelity of DNA replication because in prokaryotes, the rate of cell division is very high, and much accuracy of DNA replication and repair is needed. For most of the processes involved in DNA replication and repair, it acts as a protein-protein interaction hub. Therefore, β-clamp is essential for the reproduction as well as the survival of prokaryotes. In spite of having similar function, there is no sequence homology between prokaryotic β-clamp and eukaryotic PCNA. So, the potential drugs that target β-clamp of prokaryotes would be relatively safe for use in humans.

Out of five shortlisted drug molecules 6-Nitroindazole (C2), (S)-Carprofen (C3), and 5-Nitroindole (C4) displayed poor inhibitory activity on the growth of *H. pylori*, which may have been due to their inability to diffuse into the pathogen. Drug 5-chloroisatin (C1) and 3, 4-difluorobenzamide (C5) showed significant inhibition in μM range against *H. pylori* in our experimental condition. However, these inhibitors showed inhibition in the mM range on *E. coli* [18]. Interestingly, the sequence comparison of both proteins Hpβ-clamp and Ecβ-clamp showed an identity of 23% with 44% similarity. The inhibitor-binding site is homologous in Hpβ-clamp and Ecβ-clamp, but it is not identical. The binding of 5-chloroisatin differ clearly by one hydrogen bond, in *E. coli* b-clamp Thr172 does form H-bond while equivalent residue Thr173 in *H. pylori* does not form H-bond and interactions are dominated by hydrophobic interactions. Interestingly 3, 4-difluorobenzamide interactions are dominated by hydrophobic interactions in both structures, orientation of the molecule and interacting residues are different without any specific notable interaction. When we looked at several physio-chemical properties of these molecules (Table S1), polar surface and relative free binding energy values for 5-chloroisatin (C1) and 3, 4-difluorobenzamide (C5) showed contrasting or significantly different values from other molecules, suggesting that these parameters may play an important role in *H. pylori* targeting. Moreover, these molecules may also bind to any other targets in the organism and show better efficiency.

Overall, these results suggest that an inhibitor of a protein in one pathogen may act differently towards the orthologous protein in a different pathogen even if the orthologous proteins share a similar structure. The reason might be due to differential uptake of various drugs/inhibitors in the pathogen. It is, therefore, essential to design and test drugs against the specific pathogen of interest rather than solely relying on drugs that work on other pathogens.

## Figures and Tables

**Table 1:** Crystallographic data and refinement statistics for *H. pylori* β-clamp in complex with various inhibitors.

Data Collection			
	β-clamp Complexed with 5-chloroisatin	β-clamp Complexed with Carprofen	β-clamp Complexed with 3,4-difluorobenzamide
Space group	P21	C2	C2
Cell dimensions			
a,b,c (Å)	82.1, 65.4, 88.8	89.9, 66.4, 82.8	90.0, 66.3, 82.7
α,β,γ (deg.)	90.0, 115.7, 90.0	90.0, 115.5, 90.0	90.0, 115.4, 90.0
R_sym_ (highest resolution range)	5.0 (43.8)	5.1 (21.6)	5.5 (42.1)
Completeness (highest resolution range)	91.1 (89.0)	98.5 (79.7)	98.8 (90.9)
Mean I/σ	20.2	38.8	33.17
**Refinement**			
Resolution range (Å)	50.0–2.3	50.0–1.97	50.0–2.07
Rwork/Rfree	22.7/28.5	20.6/24.3	21.3/25.2
Number of atoms			
Protein	5588	3021	2883
Water	57	55	92
R.m.s. deviation			
Bond angles (deg.)	1.78	1.16	1.98
Bond lengths (Å)	0.015	0.005	0.007
Mean B value (Å^2^)	50.3	50.8	44.5

## Supplementary Materials

The following are available online at www.mdpi.com/2079-6382/7/1/5/s1, Figure S1: Surface representation of co-crystal structure of Hpβ-clamp monomer with ligase peptide and various inhibitors. Figure S2: Structural alignments of Hpβ-clamp-inhibitor co-complexes with the Hpβ-clamp-inhibitor docking models. Table S1: The list of *E. coli* β-clamp inhibitors along with their structures, docking scores and binding energy values with Hp β-clamp. Table S2: Table showing residues of Hpβ-clamp that interact with ligase peptide and its inhibitor molecules. Also the corresponding residues of Ecβ-clamp that are involved in binding with inhibitors have been shown.
