# Supplementary material for: Screening of E. coli β-clamp Inhibitors Revealed that Few Inhibit Helicobacter pylori More Effectively: Structural and Functional Characterization
Source: Antibiotics (Basel). 2018 Jan 11;7(1):5. doi: 10.3390/antibiotics7010005 (PMC5872116; doi:10.3390/antibiotics7010005)
Supplement: Supplementary file 1 [file antibiotics-07-00005-s001.pdf]

antibiotics

MDPI

Article

Screening of *E. coli* β-clamp Inhibitors Revealed that Few Inhibit *Helicobacter pylori* More Effectively: Structural and Functional Characterization

Preeti Pandey [1,2], Vijay Verma [3,4], Suman Kumar Dhar [3] and Samudrala Gourinath [1,*]

[1] School of Life Sciences, Jawaharlal Nehru University, New Delhi 110067, India; preet.satya@gmail.com
[2] Department of Bioscience and Biotechnology, Banasthali University, Rajasthan 304022, India
[3] Special Centre for Molecular Medicine, Jawaharlal Nehru University, New Delhi 110067, India; vijayscmmjnu@gmail.com (V.V.); skdhar2002@yahoo.co.in (S.K.D.)
[4] Department of Microbiology, Central University of Rajasthan, Kishangarh 305801, India
* Correspondence: sgourinath@jnu.ac.in; Tel.: +91-011-26704513

Received: 10 November 2017; Accepted: 10 January 2018; Published: 11 January 2018

Supplementary Materials

Table S1. The list of *E.coli* β-clamp inhibitors along with their structures, docking scores and binding energy values with Hp β-clamp.

S.No.	Name of Screened Inhibitors	Notation	Structure	Docking Score	Binding energy (kcal/mol)	Polar surface *	Relative free binding energy Kcal/mol *
1.	5-chloroisatin	C1	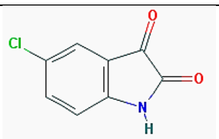	-6.88	-6.93	46.17	3.25
2.	6-Nitroindazole	C2	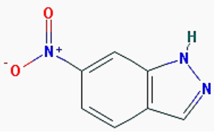	-6.88	-6.79	74.5	0.52766
3.	(S)-Carprofen	C3	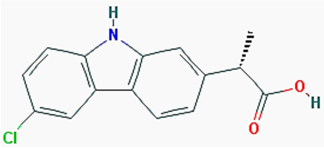	-6.03	-7.91	53.09	0.40349
4.	5-Nitroindole	C4	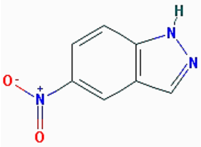	-6.88	-6.88	61.61	0.52766

antibiotics MDPI

5.	3,4-difluorobenza-mide	C5	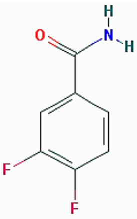	-4.64	-6.42	43.09	1.0849

* These parameters were calculated using Data warrior tool of the webserver www.openmolecules.org

Table S2. Table showing residues of Hpβ-clamp that interact with ligase peptide and its inhibitor molecules. Also the corresponding residues of Ecβ-clamp that are involved in binding with inhibitors have been shown.

S.No	Ligase peptide interact-ing residues of Hpβ-clamp	5-chloroi-satin interact-ing residues of Hpβ-clamp	5-chloroi-satin interact-ing residues of Ecβ-clamp	Carprofen interacti-ng residues of Hpβ-clamp	Carprofen interacting residues of Ecβ-clamp	3,4-difluorob-enzamide interacting residues of Hpβ-clamp	3,4-difluor-obenza-mide interact-ing residues of Ecβ-clamp
1.	-	-	-	Lys151	Arg152	Lys151	-
2.	Thr173	-	Thr172	-	Thr172	-	Thr172
3.	Thr175	Thr175	Gly174	Thr175	Gly174	Thr175	Gly174
4.	-	-	Pro242	Pro243	Pro242	Pro243	Pro242
5.	Ile248	Ile248	Val247	Ile248	Val247	Ile248	Val247
6.	Met370	-	-	Met370	Met362	Met370	-
7.	Leu368	Leu368	-	Leu368	-	Leu368	-
8.	Lys176	Lys176	His175	-	-	-	-
9.	Pro347	-	-	-	-	-	-
10.	Leu178	Leu178	-	-	-	-	Leu177
11.	Met369	-	-	-	-	-	-
12.	Arg177	Arg177	Arg176	-	-	-	-
13.	-	-	-	-	Tyr154	-	-

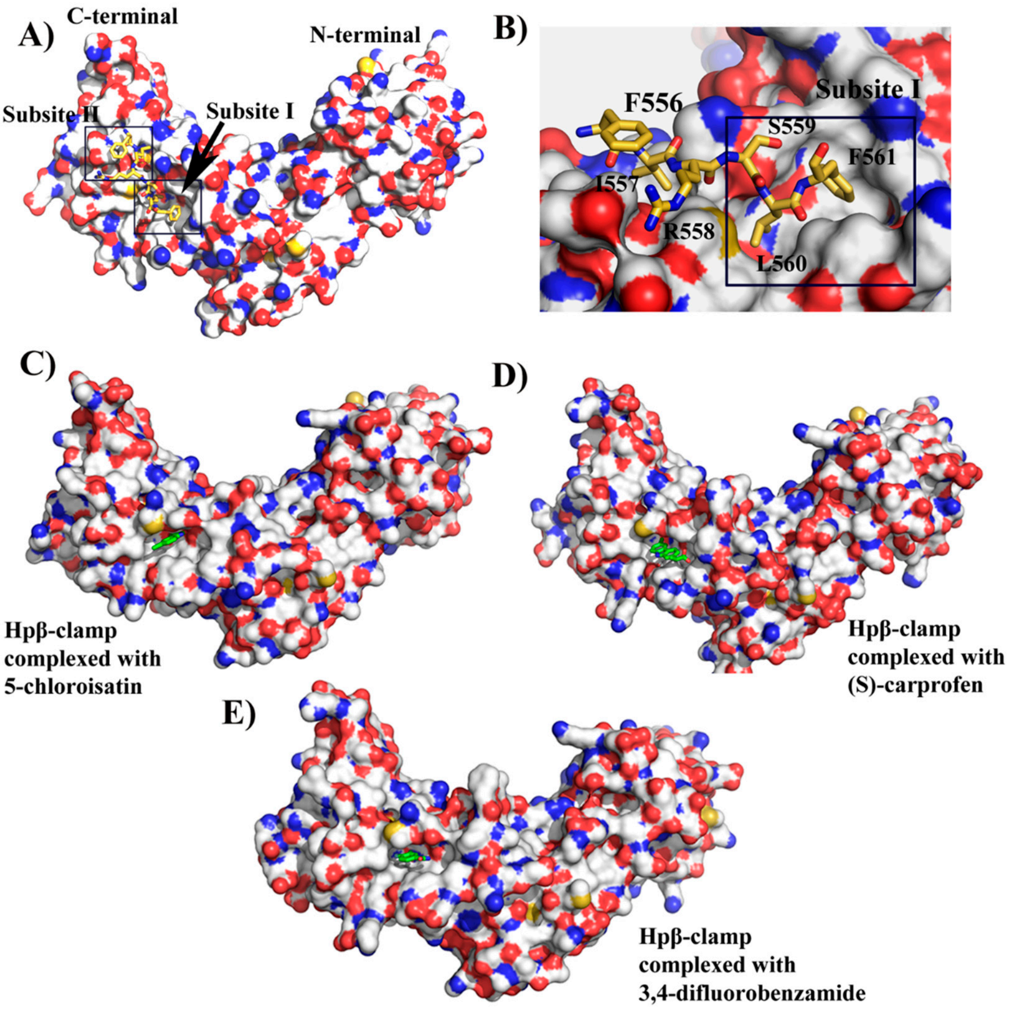

Figure S1. Surface representation of co-crystal structure of Hpβ-clamp monomer with ligase peptide and various inhibitors. (A) Hpβ-clamp bound to peptide from HpDNA ligase. The rectangular regions in the figure show the protein-interacting sites of Hpβ-clamp, i.e., subsite I and subsite II, encompassing the region between domain II and domain III. These subsites were observed to be hydrophobic. (B) Enlarged view of ligase peptide-binding region of Hpβ-clamp. The last two residues of the peptide, L560 and F561, were found to interact with and fit deep into the cleft of subsite I. (C-E) Surface representations of inhibitor-bound Hpβ-clamps, with the inhibitors being (C) 5-chloriosatin, (D) (S)-carprofen, (E) 3, 4-difluorobenzamide. All of these ligands bound to the hydrophobic cleft of subsite I where DNA ligase has also been found to bind.

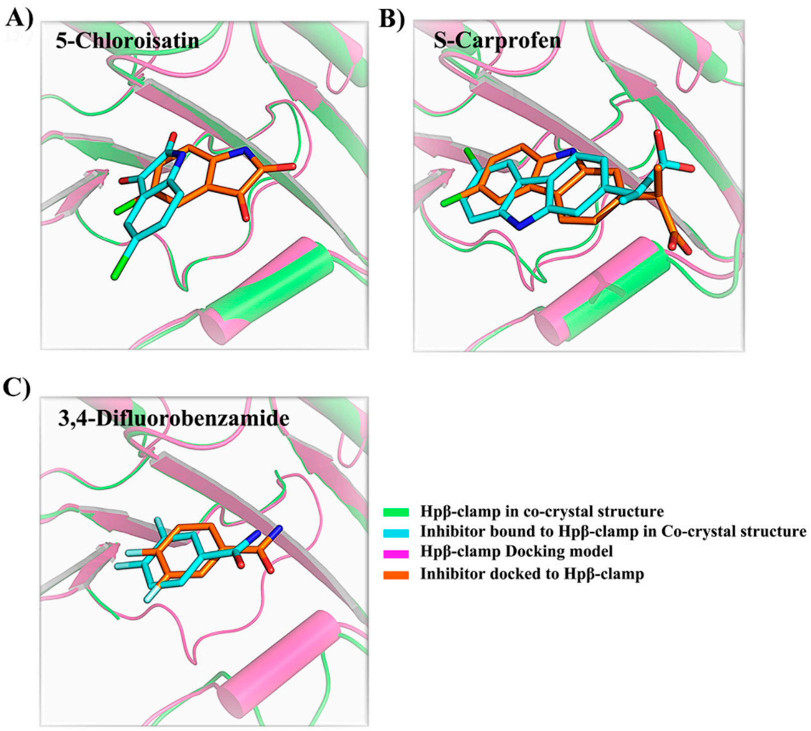

Figure S2. Structural alignments of Hpβ-clamp-inhibitor co-complexes with the Hpβ-clamp-inhibitor docking models. (A) Superimposition of the structure of the inhibitor 5-chloroisatin (orange) docked into Hpβ-clamp (pink) on the corresponding co-crystal structure (green and cyan for Hpβ-clamp and inhibitor, respectively). The orientation of the inhibitor in the docked structure was observed to differ considerably from that in the co-crystal structure. (B) Superimposition of the structure of the inhibitor (S)-carprofen (orange) docked into Hpβ-clamp (pink) on the corresponding co-crystal structure (green and cyan for Hpβ-clamp and inhibitor, respectively). Note that at the resolution of the data, the orientation of this planar inhibitor molecule cannot be distinguished from that rotated by 180 degrees. (C) Superimposition of the structure of the inhibitor 3, 4-difluorobenzamide (orange) docked into Hpβ-clamp (pink) on the corresponding co-crystal structure (green and cyan for Hpβ-clamp and inhibitor, respectively). The orientation of the inhibitor in the docked structure and that in the co-crystallized structure were observed to be similar.
